# Assessment of outcomes and novel immune biomarkers in metaplastic breast cancer: a single institution retrospective study

**DOI:** 10.1186/s12957-019-1780-8

**Published:** 2020-01-14

**Authors:** Evan Morgan, Anupama Suresh, Akaansha Ganju, Daniel G. Stover, Robert Wesolowski, Sagar Sardesai, Anne Noonan, Raquel Reinbolt, Jeffrey VanDeusen, Nicole Williams, Mathew A. Cherian, Zaibo Li, Gregory Young, Marilly Palettas, Julie Stephens, Joseph Liu, Amanda Luff, Bhuvaneswari Ramaswamy, Maryam Lustberg

**Affiliations:** 1 Stefanie Spielman Comprehensive Breast Cancer, The Ohio State University, Columbus, OH USA; 2 Division of Medical Oncology, Comprehensive Cancer Center, The Ohio State University Medical Center, Columbus, OH USA; 3 Department of Internal Medicine, Riverside Methodist Hospital, Columbus, Ohio USA; 4 Department of Pathology, The Ohio State University Medical Center, Columbus, OH USA; 5 Center for Biostatistics, Department of Biomedical Informatics, The Ohio State University, Columbus, OH USA

**Keywords:** Metaplastic breast cancer, Clinical outcomes, Distant disease-free survival, Overall survival, Triple-negative breast cancer, Immune markers

## Abstract

**Background:**

Metaplastic breast cancer remains poorly characterized given its rarity and heterogeneity. The majority of metaplastic breast cancers demonstrate a phenotype of triple-negative breast cancer; however, differences in clinical outcomes between metaplastic breast cancer and triple-negative breast cancer in the era of third-generation chemotherapy remain unclear.

**Methods:**

We compared the clinical outcomes between women with metaplastic breast cancer and women with triple-negative breast cancer diagnosed between 1994 and 2014. Metaplastic breast cancer patients were matched 1:3 to triple-negative breast cancer patients by stage and age at diagnosis. Distant disease-free survival (DDFS) and overall survival (OS) were estimated using Kaplan Meier methods and Cox proportional hazard regression models. Immune checkpoint markers were characterized by immunohistochemistry in a subset of samples.

**Results:**

Forty-four metaplastic breast cancer patients (stage I 14%; stage II 73%; stage III 11%; stage IV 2%) with an average age of 55.4 (± 13.9) years at diagnosis. Median follow-up for the included metaplastic breast cancer and triple-negative breast cancer patients (*n* = 174) was 2.8 (0.1–19.0) years. The DDFS and OS between matched metaplastic breast cancer and triple-negative breast cancer patients were similar, even when adjusting for clinical covariates (DDFS: HR = 1.64, *p* = 0.22; OS: HR = 1.64, *p* = 0.26). Metaplastic breast cancer samples (*n* = 27) demonstrated greater amount of CD163 in the stroma (*p* = 0.05) and PD-L1 in the tumor (*p* = 0.01) than triple-negative breast cancer samples (*n* = 119), although more triple-negative breast cancer samples were positive for CD8 in the tumor than metaplastic breast cancer samples (*p* = 0.02).

**Conclusions:**

Patients with metaplastic breast cancer had similar outcomes to those with triple-negative breast cancer based on DDFS and OS. The immune checkpoint marker profile of metaplastic breast cancers in this study may prove useful in future studies attempting to demonstrate an association between immune profile and survival.

## Introduction

Metaplastic breast cancer (MBC) is a very rare type of invasive breast cancer in which the original cell type, usually glandular epithelium, differentiates into either epithelial and/or mesenchymal cell types with glandular and non-glandular components [1, 2]. According to the World Health Organization (WHO), MBC includes several subtypes, including low-grade adenosquamous carcinoma, fibromatosis-like metaplastic carcinoma, squamous cell carcinoma, spindle cell carcinoma, mixed metaplastic carcinoma, myoepithelial carcinoma, and metaplastic carcinoma with mesenchymal differentiation—notably chondroid, osseous, and other types of mesenchymal differentiation [3]. MBC accounts for 0.2–5% of all breast cancers, yet the lack of an accepted definition may contribute to the varying prevalence rates [4].

The majority of MBCs are triple-negative, which is defined as breast tumors that are negative for the estrogen receptor, progesterone receptor, and do not overexpress HER2/neu. Furthermore, an immunohistochemical panel showed that 93.8% of MBCs were basal-like, the most common subset of triple-negative breast cancer (TNBC) [5]. MBCs tend to have a large tumor size, rapid growth, and less axillary lymph node involvement [6, 7]. Although there is less axillary node involvement, like soft tissue sarcomas, MBCs are more prone to hematogenous dissemination and have a poor prognosis [8, 9]. While this may be in part due to the preponderance of the TNBC phenotype among MBCs, one prior study comparing MBC and triple-negative invasive ductal cancers showed that MBCs had worse prognosis relative to TNBCs, with shorter disease-free survival (DFS) in patients with nodal metastasis treated with adjuvant chemotherapy [10].

Patients with MBC are significantly more likely to receive chemotherapy compared to patients with invasive carcinoma of no special type (IC-NST) [11], the most common histologic type of breast cancer. However, MBC has demonstrated resistance to traditional forms of chemotherapy [9, 12]. It is important to assess how the treatment of these cancers has evolved over time, given disparities in clinical outcomes between MBC and other TNBC. Notably, the rarity of MBC leads to difficulty in identifying effective treatment strategies through clinical trials. Although evidence from randomized controlled trials supporting the use of neoadjuvant or adjuvant chemotherapy for MBC is limited, the majority of patients with MBC receive chemotherapy, given the high risk of relapse and poor prognosis [13].

In recent years, immunotherapeutic agents that target components of tumor microenvironments (TMEs) have shown potential for the treatment of TNBC; however, clinical trials are still ongoing[14–16]. The interaction between the immune regulatory proteins, programmed cell death-1 (PD-1) and programmed death ligand-1 (PD-L1), is of particular interest as it has been shown to facilitate tumor progression through inactivation of tumor-infiltrating lymphocytes (TILs) [17, 18]. High levels of the immune regulatory protein PD-L1 are seen in both TNBC and MBC, though PD-L1 expression is higher in MBCs [19, 20]. Clinical trials utilizing PD-L1 and PD-1 inhibitors in the treatment of TNBC are ongoing, with results demonstrating response to immunotherapy as first-line therapy or in combination with chemotherapy [21–24]. Infiltration of cytotoxic T cells (CD8^+^ T cells), which are negatively regulated by PD-1, in residual tumors is associated with better clinical outcomes in TNBC treated with neoadjuvant chemotherapy [25], though this relationship has not yet been examined in MBC. CD163, a scavenger receptor for the hemoglobin-haptoglobin complex, is a marker for alternatively activated (M2) polarized macrophages [26]. CD163^+^ tumor-associated macrophage (TAM) infiltration in tumor stroma is also of clinical interest as it is strongly associated with TNBC and is associated with poorer survival in TNBC with low TIL levels [27–29], though the role of CD163^+^ TAM infiltration in MBC has not been examined.

Given its rare occurrence and heterogeneous classification, MBC remains poorly characterized. While most MBC is triple-negative and treated as such, the differences in outcomes between MBC and TNBC led us to perform a 1:3 matched comparison to evaluate survival outcomes in the era of third-generation chemotherapy. In this retrospective study, we aimed to compare distant disease-free survival (DDFS) and overall survival (OS) between patients with MBC treated at The Ohio State University Comprehensive Cancer Center (OSUCCC–James) and those with non-metaplastic TNBC. Further, we assessed and compared immune marker expression in the TME of MBC and TNBC with a goal to identify immune markers which can be potential targets and provide prognostic value.

## Methods

### Study design

Patients treated at The Ohio State University Comprehensive Cancer Center–Arthur G. James Cancer Hospital and Richard J. Solove Research Institute between January 1, 1994, and December 31, 2014, with a diagnosis of TNBC or MBC were eligible for this retrospective study. Following IRB approval (OSU 2015C0135), the list of patients fulfilling this eligibility criterion, i.e., any woman with a diagnosis of TNBC or MBC between January 1, 1994, and December 31, 2014, was obtained from Ohio State University Wexner Medical Center and James Cancer Registry. Patients with ICD-O-3 Histology Code associated with “Metaplastic carcinoma, NOS” were identified through the OSUCCC–James Cancer Registry, and MBC diagnosis was confirmed through pathology report review prior to inclusion in the study. Patients with ICD 9 code diagnosis of breast cancer (174.0–174.9) and pathology negative for estrogen, progesterone and HER2 receptor overexpression were considered to have TNBC. The OSUCCC–James Cancer Registry determined receptor status through the review of individual pathology reports utilizing the guidelines from the College of American Pathologists (CAP) and the American Society of Clinical Oncology (ASCO) that were available at the time of diagnosis. Patient charts with incomplete data were excluded from the study. Each MBC patient was matched with three non-metaplastic TNBC patients based on stage and age at diagnosis [30]. Stage (I–IV) was required to be identical and age at diagnosis was restricted to within 10 years.

### Data collection

The following data were extracted from patients’ medical records: patient’s age at time of diagnosis, race, ethnicity, height, weight, stage, biomarker profiles (ER, PR, and HER2) of the tumor, therapy modality (surgery, chemotherapy type and regimen, and radiotherapy), and duration, as well as survival data including distant disease-free survival and overall survival.

### Multi-color multiplex immunohistochemistry and assessment of checkpoint immune system

Multi-color multiplex immunohistochemical (IHC) assays capable of demonstrating co-localization of PD-L1 with CD8 and CD163 were performed on fresh-cut whole sections from patients’ resection specimens on an Autostainer BenchMark XT platform (Ventana Medical Systems, Inc., Tucson, AZ; Ventana) according to the manufacturer’s recommendation. The antibodies used were as follows: for PD-L1 clone SP263, rabbit, Ventana; for CD8 clone SP57, rabbit, Ventana; for CD163 clone MRQ26, mouse, Ventana. SP263 is the only available PD-L1 antibody in the multiplex IHC assay. PD-L1 signal was detected using iVIEW DAB IHC Detection Kit (Ventana) with brown color, CD8 signal was detected using iVIEW HRP Green IHC Detection Kit (Ventana) with green color and CD163 signal was detected using iVIEW Fast Red IHC Detection Kit (Ventana) with red color. IHCs were evaluated by a pathologist (ZL) using a semi-quantitative approach for the entire tumor and tumor-surrounding areas under a microscope. Positive (previously confirmed PD-L1-positive breast carcinoma specimen) and negative (previously confirmed PD-L1-negative breast carcinoma specimen) controls were included for each batch of IHCs. Membranous PD-L1 staining in tumor cells or immune cells was considered as a specific staining. A positive PD-L1 expression among tumor cells was defined as any membranous staining in ≥1% of tumor cells in order to maximize the assay sensitivity for PD-L1-positive cases [31, 32]. The following parameters were assessed: PD-L1 expression in tumor cells, PD-L1 expression in immune cells, CD8+ immune cells within the tumor, CD8+ immune cells within the stroma, CD163+ macrophages within the tumor, and CD163+ macrophages within the stroma. The cut-off percentage for CD8+ cells and CD163+ cells was set at 10% [32].

### Statistical analysis

Analyses focused on the comparisons between the age at initial diagnosis and stage-matched MBC and TNBC patients. The primary endpoints of this study were DDFS and OS. The DDFS period was defined as the time from diagnosis to the date of the first observation of distant disease recurrence, while the OS period was defined as the time from diagnosis to death or censoring. An exploratory endpoint was to define the expression of immune markers in the TME of samples from primary MBC. A comparison of clinicopathological and treatment characteristics was achieved using a two-sample *t* test for continuous variables and a Fisher’s exact test for categorical variables. Kaplan-Meier methods were used to estimate survival curves for OS and DDFS for the two groups. Patients diagnosed as stage IV were eliminated from analyses of DDFS. The log-rank test was used to compare the curves. Cox proportional hazard regression models were used to examine DDFS and OS between MBC and TNBC groups while adjusting for additional relevant clinical covariates such as age at initial diagnosis, stage, use of chemotherapy and radiotherapy, and lymph node involvement.

## Results

### Patient characteristics

Our review of medical records identified 382 patients who were eligible for this study. Of these, 44 had MBC while the remaining 338 patients had non-metaplastic TNBC. Each MBC patient was matched with three TNBC patients based on age and stage except for one 93-year-old MBC patient who had only one age- and stage-matched TNBC patient. Hence, 130 TNBC patients were included in the study. Median follow-up, defined as the time from diagnosis to death or censoring, for the included MBC and TNBC patients (*n* = 174) was 2.8 (0.1–19.0) years, with only 8 patients followed for more than 10 years.

The demographic data of this population are listed in Table 1. The average age for MBC patients was 55.4 (± 13.9) years at diagnosis. The majority of patients with MBC presented as stage II breast cancer (72.7%). Fewer MBC patients were node-positive at the presentation when compared to TNBC, though the difference was not significant (29.5% vs 46.2%, *p* = 0.08).

**Table 1:** Demographic and clinical data by group

Variable	Level	TNBC (*n* = 130)	MBC (*n* = 44)	Total	*p* value
Age at initial diagnosis	Mean (SD)	54.6 (12.8)	55.4 (13.9)	54.8 (13.0) [*n* = 174]	0.7495
Race	White	108 (83.1%)	40 (90.9%)	148 (85.1%)	0.2451
	Black	14 (10.8%)	4 (9.1%)	18 (10.3%)	—
	Other	8 (6.2%)	0 (0.0%)	8 (4.6%)	—
Hispanic ethnicity	Yes	3 (2.3%)	0 (0.0%)	3 (1.8%)	1.0000
Positive nodes	Yes	60 (46.2%)	13 (29.5%)	73 (42.0%)	0.0765
Stage	I	16 (12.3%)	6 (13.6%)	22 (12.6%)	0.9812
	II	96 (73.8%)	32 (72.7%)	128 (73.6%)	—
	III	15 (11.5%)	5 (11.4%)	20 (11.5%)	—
	IV	3 (2.3%)	1 (2.3%)	4 (2.3%)	—
ER status	Positive	0 (0.0%)	4 (9.1%)	4 (2.3%)	0.0037
	Negative	130 (100.0%)	40 (90.9%)	170 (97.7%)	—
PR status	Positive	0 (0.0%)	4 (9.1%)	4 (2.3%)	0.0037
	Negative	130 (100.0%)	40 (90.9%)	170 (97.7%)	—
HER2 status	Positive	0 (0.0%)	1 (2.3%)	1 (0.6%)	0.0146
	Negative	130 (100.0%)	43 (97.7%)	170 (98.3%)	—

*p* values from a two-sample *t* test for continuous variables and a Fisher’s exact test for categorical variables. Abbreviations: *ER* = estrogen receptor; *HER2* = human epidermal growth factor receptor 2; *MBC* = metaplastic breast cancer; *PR* = progesterone receptor; *SD* = standard deviation; *TNBC* = triple-negative breast cancer

### Treatment

The details of the treatment modalities are summarized in Table 2. Overall treatment modalities were similar, including rates of type of surgery (lumpectomy versus mastectomy), radiation, and receipt of any type of chemotherapy. Among specific agents, taxanes were used less frequently for the treatment of MBC patients compared to non-metaplastic TNBC patients (70.5% vs 85.4%, *p* = 0.0411). Among MBC patients, there were three patients with estrogen receptor (ER)-positive and progesterone receptor (PR)-negative (ER+/PR−) MBC (≤ 10% cells ER+), three patients with ER-negative and PR-positive (ER−/PR+) MBC (≤ 10% cells PR+), and one patient with ER+/PR+ MBC (< 10% cells ER+/PR+). Two patients with ER+/PR− MBC received a form of anti-estrogen therapy, while the third had a previous history of bilateral oophorectomy. Two patients with ER−/PR+ MBC received a form of anti-estrogen therapy, while the third patient did not. One patient with HER2 positive (HER2+) MBC received anti-HER2 therapy.

**Table 2:** Treatment data by group

Variable	Level	TNBC (*n* = 130)	MBC (*n* = 44)	Total	*p* value
Local therapy	None	4 (3.1%)	1 (2.3%)	5 (2.9%)	0.8203
	Complete mastectomy	33 (25.4%)	15 (34.1%)	48 (27.6%)	—
	Lumpectomy	2 (1.5%)	1 (2.3%)	3 (1.7%)	—
	Radiation therapy	2 (1.5%)	0 (0.0%)	2 (1.1%)	—
	Complete mastectomy + radiation therapy	37 (28.5%)	13 (29.5%)	50 (28.7%)	—
	Lumpectomy + radiation therapy	52 (40.0%)	14 (31.8%)	66 (37.9%)	—
Radiation	Yes	91 (70.0%)	27 (61.4%)	118 (67.8%)	0.3509
Breast conserving surgery	Yes	54 (41.5%)	15 (34.1%)	69 (39.7%)	0.4763
Chemotherapy in first year following diagnosis	Yes	119 (91.5%)	38 (86.4%)	157 (90.2%)	0.3782
Anthracycline therapy	Yes	104 (80.0%)	34 (77.3%)	138 (79.3%)	0.6731
Platinum therapy	Yes	17 (13.1%)	6 (13.6%)	23 (13.2%)	1.0000
Taxane therapy	Yes	111 (85.4%)	31 (70.5%)	142 (81.6%)	0.0411

Chemotherapy regimens in which at least 5 patients received were included. *p* values from a two-sample *t* test for continuous variables and a Fisher’s exact test for categorical variables. Abbreviations: *MBC* = metaplastic breast cancer, *TNBC* = triple-negative breast cancer

### Clinical outcomes

**Fig. 1:**
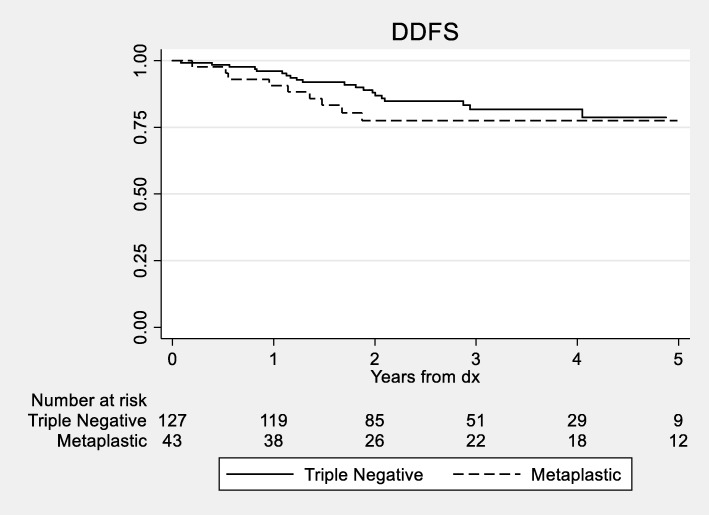
Kaplan-Meier curves for distant disease-free survival between patients with metaplastic and triple-negative breast cancer (*p* = 0.35). Only the first 5 years of survival time is displayed, as few patients (*n* = 21) had follow-up past 5 years. Abbreviations: DDFS = distant disease-free survival; *dx* = diagnosis

**Fig. 2:**
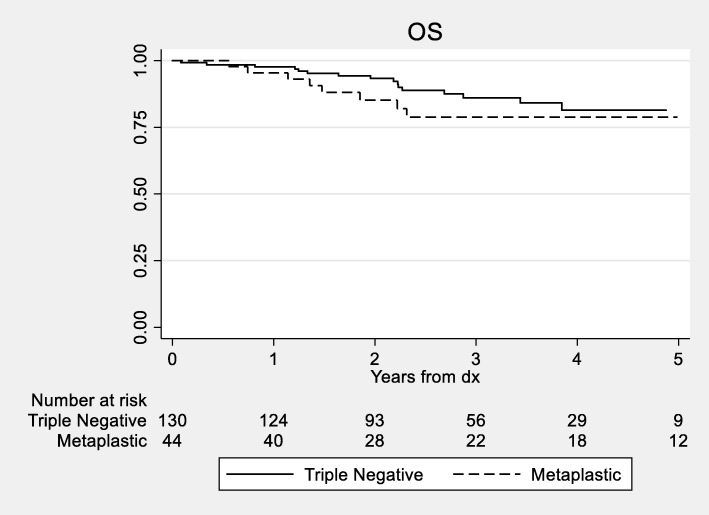
Kaplan-Meier curves for overall survival between patients with metaplastic and triple-negative breast cancer (*p* = 0.32). Only the first 5 years of survival time is displayed, as few patients (*n* = 21) had follow-up past 5 years. Abbreviations: OS = overall survival; *dx* = diagnosis

The median DDFS for MBC patients was 10.9 years vs 13.7 years for non-metaplastic TNBC patients. We did not detect a statistically significant difference in 3- and 5-year DDFS between the two groups with the DDFS for MBC patients of 77.5% and 77.5% vs 81.7% and 78.7% (log rank *p* = 0.35; Fig. 1) for non-metaplastic TNBC patients, respectively. There were too few deaths among the non-metaplastic patients to reach the median overall survival in the TNBC group for OS. We did not detect a statistically significant difference in OS between the groups (log-rank *p* = 0.32, Fig. 2). Estimates of 3- and 5-year OS were 78.9% and 78.9%.vs 86.1% and 81.4% for MBC and TNBC patients, respectively.

Multivariate analyses (Table 3) showed that there was no significant difference in DDFS (HR = 1.64, 95% CI = 0.75–3.58, *p* = 0.22) and OS (HR = 1.64, 95% CI = 0.69–3.90, *p* = 0.26) for MBC vs TNBC patients, when adjusting for clinical stage, nodal status, age, use of any chemotherapy, and radiotherapy. Patients with nodal metastases showed decreased likelihood of DDFS (HR = 4.32, 95% CI = 1.66–11.29, *p* = 0.003) and a trend towards worse OS (HR = 2.62, 95% CI = 0.88–7.82, *p* = 0.08). Stage was a significant predictor of DDFS (*p* = 0.03) with stage I (HR = 0.33, 95% CI = 0.06–1.83) and II (HR = 0.32, 95% CI = 0.14–0.74) patients having longer survival than stage III. Results for OS were similar (*p* = 0.002) with stage I (HR = 0.06, 95% CI = 0.01–0.54) and II (HR = 0.07, 95% CI = 0.02–0.28) patients showing greater survival. No other variables had a significant effect on DDFS or OS following multivariate analysis.

**Table 3:** Multivariate Cox model for DDFS (*n* = 170) and OS (*n* = 174)

Outcome	MV analysis of DDFS with nodal status		MV analysis of OS with nodal status	
	HR (95% CI)	*p* value	HR (95% CI)	*p* value
Age at initial diagnosis	1.01 (0.98, 1.04)	0.6608	1.03 ( 0.99, 1.07)	0.1252
Chemotherapy given in the first year following diagnosis	0.42 (0.11, 1.65)	0.2129	0.71 ( 0.16, 3.21)	0.6552
Metaplastic diagnosis	1.64 (0.75, 3.58)	0.2159	1.64 ( 0.69, 3.90)	0.2597
Positive nodes	4.32 (1.66, 11.29)	0.0028	2.62 ( 0.88, 7.82)	0.0844
Radiotherapy given	0.58 (0.23, 1.50)	0.2643	0.55 ( 0.20, 1.49)	0.2384
Stage 1	0.33 (0.06, 1.83)	0.0287	0.06 ( 0.01, 0.54)	0.0021
Stage 2	0.32 (0.14, 0.74)	—	0.07 ( 0.02, 0.28)	—
Stage 3	Ref	—	0.24 ( 0.06, 0.93)	—
Stage 4	—	—	Ref	—

Abbreviations: *CI* = confidence interval; *DDFS* = distant disease-free survival; *HR* = hazard ratio; *MV* = multivariate; *OS* = overall survival

Given differences in taxane use, we examined the use of taxane chemotherapies via an additional multivariate analysis using a categorical variable (no chemotherapy, taxane, and non-taxane) while adjusting for age, use of radiation therapy, nodal status, and metaplastic diagnosis. A majority (90%, *n* = 142) of the patients who received chemotherapy received a taxane, and only a few patients (n = 15) received non-taxane therapy. We observed no significant effect on DDFS (*p* = 0.56) or OS (*p* = 0.80) between taxane and non-taxane use.

### Immune checkpoint marker expression

**Fig. 3:**
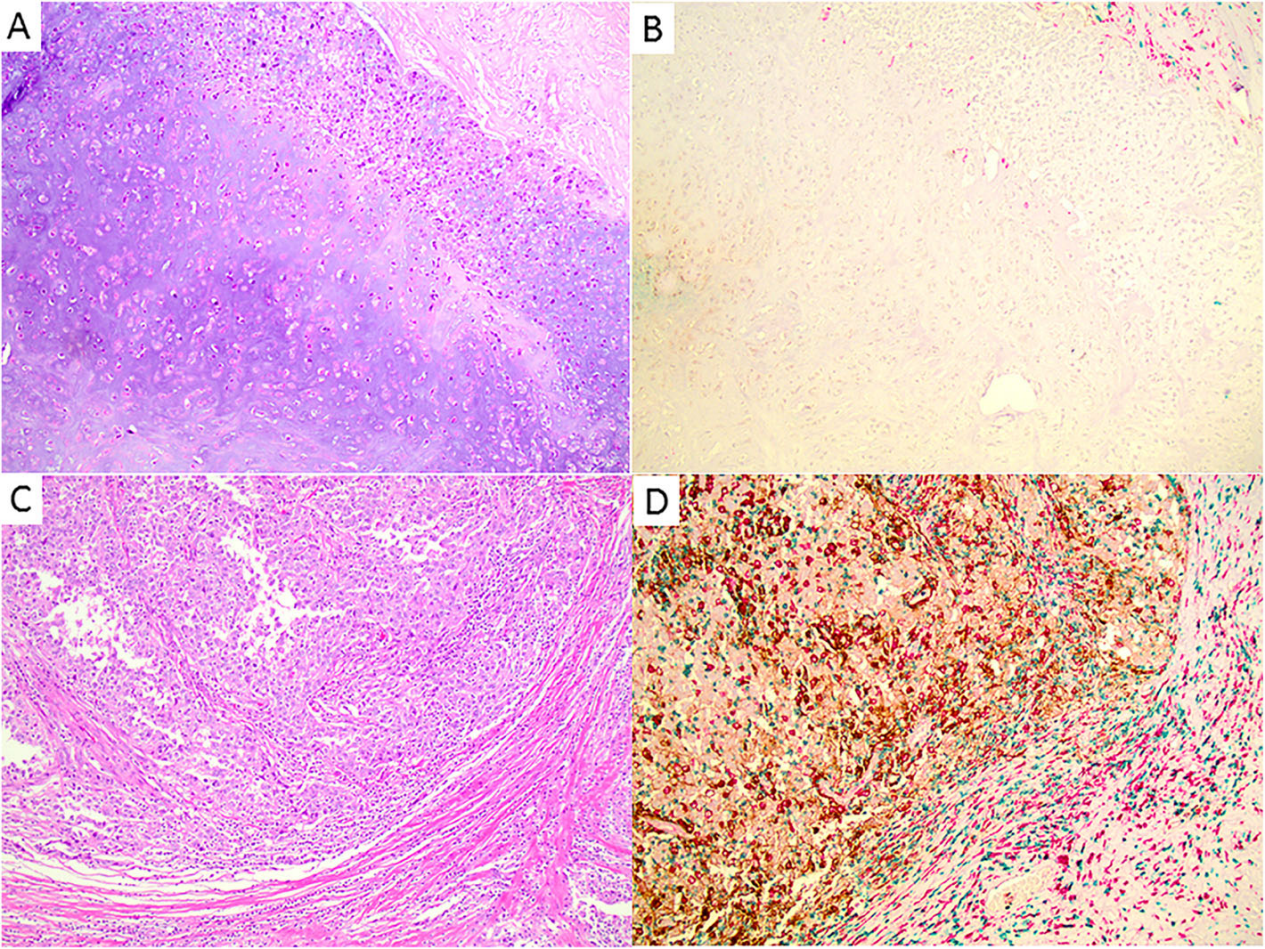
Representative images of different immune reaction and PD-L1 expressions in two invasive metaplastic breast carcinomas, as detected with anti-PD-L1 multiplex immunohistochemistry (anti-CD8 in green, anti-CD163 in red, and anti-PD-L1 in brown). **a**, **b** One invasive metaplastic carcinoma with no PD-L1 expression, only scattered CD163^+^ cells and very rare CD8^+^ cytotoxic T-cells in the peritumoral stroma. **c**, **d** One invasive metaplastic carcinoma with strong PD-L1 expression in tumor cells and stromal cells, diffuse CD163^+^ cells and CD8^+^ cytotoxic T-cells in tumoral stroma and peritumoral stroma. Magnification: × 100

The expression of various immune markers within MBC is summarized in Table 4. PD-L1 expression on tumor cells was detected in 29.6% of MBC samples (*n* = 27). Immune marker testing was not possible for 38.6% of cases (*n* = 17) lacking sufficient tissue for staining (blocks or unstained slides). A separate cohort of TNBC patients (*n* = 119) was used to compare tissue immune markers with MBC samples, seen in Table 4. More MBC samples demonstrated CD163^+^ cells in the stroma (96.3% vs. 79.8%, *p* = 0.0468) and positive PD-L1 expression in tumor cells (29.6% vs. 10.1%, *p* = 0.0133) compared to TNBC samples. However, significantly more TNBC samples demonstrated high levels of CD8^+^ immune cells in the tumor compared to MBC samples (44.5% vs. 18.5%, *p* = 0.0158). Figure 3 shows images of the different immune markers and PD-L1 expressions in two metaplastic breast carcinomas. The immune marker profile of our MBC samples is analogous to those generated from TNBC samples in previous studies, which demonstrated associations between lower CD8, higher CD163, higher PD-L1 staining and worse prognosis [25, 28].

**Table 4:** Comparison of immune marker expression in TNBC and MBC samples

Metaplastic breast carcinoma immune checkpoint markers					
Outcome	Level	TNBC (*n* = 119)	MBC (*n* = 27)	Total	*p* value
Age at initial diagnosis	Mean (SD)	51.9 (11.8)	57.8 (15.3)	53.0 (12.7)	0.0281
CD8^+^% in stroma ≥ 10	No	25 (21.0%)	6 (22.2%)	31 (21.2%)	1.0000
	Yes	94 (79.0%)	21 (77.8%)	115 (78.8%)	0.2451
CD8^+^% in tumor ≥ 10	No	66 (55.5%)	22 (81.5%)	88 (60.3%)	0.0158
	Yes	53 (44.5%)	5 (18.5%)	58 (39.7%)	—
CD163^+^% in tumor ≥ 10	No	64 (53.8%)	14 (51.9%)	78 (53.4%)	1.0000
	Yes	55 (46.2%)	13 (48.1%)	68 (46.6%)	0.0765
CD163^+^% in stroma ≥ 10	No	24 (20.2%)	1 (3.7%)	25 (17.1%)	0.0468
	Yes	95 (79.8%)	26 (96.3%)	121 (82.9%)	—
PD-L1% in tumor ≥ 1	No	107 (89.9%)	19 (70.4%)	126 (86.3%)	0.0133
	Yes	12 (10.1%)	8 (29.6%)	20 (13.7%)	—
PD-L1% in stroma ≥ 1	No	32 (26.9%)	11 (40.7%)	43 (29.5%)	0.1665
	Yes	87 (73.1%)	16 (59.3%)	103 (70.5%)	—
PD-L1% overall ≥ 1	No	32 (26.9%)	12 (44.4%)	44 (30.1%)	0.1025
	Yes	87 (73.1%)	15 (55.6%)	102 (69.9%)	—

TNBC samples were obtained from a different cohort of patients. *p* values from a two-sample t-test for continuous variables and a Fisher’s exact test for categorical variables. Abbreviations: *MBC* = metaplastic breast cancer; *SD* = standard deviation; *TNBC* = triple-negative breast cancer

## Discussion

MBC is a rare and heterogeneous type of invasive breast cancer that subsequently lacks research demonstrating consistent direction. Moreover, MBC is historically known to have an aggressive nature with a questionable response the chemotherapy. Our single institution, retrospective study compared the clinical and histopathologic features, management, outcomes, and immune marker expression between patients with MBC and a matched cohort of patients with TNBC. We found that patients with MBC had similar outcomes to TNBC based on DDFS and OS, unlike previous publications [6, 10, 33]. Furthermore, the study identified that treatment with a taxane or anthracycline type chemotherapy was common among our patients with MBC (70.5% and 77.3%), in contrast with rates seen in a previous report [34]. Finally, the levels of immune marker staining seen in our MBC samples (lower CD8, higher CD163, and higher PD-L1) is comparable to data from previous publications using TNBC samples [25, 28].

The prognosis of MBC, when compared to TNBC, has traditionally been known to be worse, but survival outcomes have varied across numerous studies. One study examining outcomes of MBC and triple-negative IC-NST patients found no significant differences in early DFS between the two groups [10]. A large international study comparing survival outcomes between patients with MBC and those with histological grade, lymph node stage, ER, and HER2 status matched conventional no specific type/invasive ductal primary breast carcinomas revealed significantly different rates of breast cancer-specific survival (BCSS—defined as the interval between primary surgery and death); however, the difference in outcomes did not remain following the exclusion of locally advanced patients [35]. Such results are similar to our multivariate analysis that showed no significant difference in OS and DDFS between the two cohorts. However, other investigations have demonstrated unfavorable results for patients with MBC compared to those with TNBC or hormone receptor-negative IC-NST [6, 36–38]. The varying results highlight the need for additional larger retrospective studies to understand the biology and best treatment practices to treat patients with MBC.

We reviewed the literature to survey the chemotherapy types and regimens used in the treatment of MBC in comparison to TNBC and identified four studies published between 2012 and 2017, summarized in Table 5. While survival rates for the TNBC patients in our study were similar to those in the other studies (5-year DDFS = 78.7%, 5-year OS = 81.4%), all other studies demonstrated worse prognosis for patients with MBC in comparison to TNBC. Notably, only one study examined the difference in the use of chemotherapy by class between MBC and TNBC groups [34]. While taxane therapies were used more frequently among patients with MBC (70.5%) and TNBC (85.4%) in this study compared to those in Aydiner et al. (46.3% and 72.5%, respectively), taxane use was not independently associated with either survival outcome, though our sample size was limited. Nevertheless, the use of taxane therapies might account for the relatively improved survival seen among MBC patients in this study compared to other cohorts of MBC patients, although this is a retrospective observation and the prospective validation of this hypothesis is difficult due to rarity of patients with MBC.

**Table 5:** Review of studies comparing MBC and TNBC

			Outcomes (MBC vs TNBC)					
Author (year)	Sample (N)	Matching	Analysis	DFS % (*p*)	DFS HR (*p*)	OS % (*p*)	OS HR (*p*)	Chemotherapy
Lee H, et al. (2012) [31]	MBC = 67 TNBC = 520	1:8 -Stage	Univariate		3.65 (*p* < 0.001)		3.5 (*p* < 0.001)	MBC and TNBC stage I–III: -16.7% and 20.5% anthracycline- and taxane (combination)-based NACTx MBC and TNBC recurrence or stage IV: -17% anthracycline based -35% taxane based -30% capecitabine containing -17% other Distribution of regimens between MBC and TNBC (*p* = 0.280)
			Multivariate		2.53 (*p* = 0.005)		2.56 (*p* = 0.017)	
Aydiner A, et al. (2015) [32]	MBC = 54 TNBC = 51	1:1 -Clinical and pathologic features -Demographic features -Treatment modality	Univariate	PFS* 3 years 51% vs 82% (*p* = 0.013)		3 years 68% vs 94% (*p* = 0.009)		MBC and TNBC stage I–IV: - 36% and 28% anthracycline based - 2% and 8% taxane based - 48% and 64% anthracycline + taxane based - 14% and 0% other regimens Distribution of regimens between MBC and TNBC (*p* = 0.67)
			Multivariate		PFS* 0.09 (*p* < 0.001)		0.35 (*p* = 0.19)	
El Zein D, et al. (2017) [33]	MBC = 46 TNBC = 508	1:1 -Age -Stage -Grade -Treatment setting -Treatment modality	Univariate	5 years 74% vs 90% (*p* < 0.001)		5 years 65% vs 87% (*p* = 0.002)		MBC stage I–III**:** -78.6% AC/T -4.3% CMF -2.1% MAID -6.5% carboplatin based -4.3% gemcitabine based Distribution of regimens between MBC and TNBC (*p* = N/A)
			Multivariate		1.99 (*p* = 0.05)		1.50 (*p* = 0.25)	
Song Y, et al. (2013) [34]	MBC = 55 TNBC = 131	1:2 -Age -Period of diagnosis	Univariate	5 years 46% vs 60% (*p* < 0.001)		5 years 55% vs 73% (*p* < 0.001)		MBC stage I–III: -37.5% TAC adjuvant CTx -14.5% CMF adjuvant CTx -14.5% FAC adjuvant CTx -18.75% FAC→TC adjuvant CTx -8.3% TAC + cisplatin/capecitabine adjuvant CTx -6.25% TAC + capecitabine/vinorelbine adjuvant CTx Distribution of regimens betweenMBC and TNBC (*p* = N/A)
			Multivariate	Not performed vs TN-IDC		Not performed vs TN-IDC		
Current study	MBC = 44 TNBC = 130	1:3 -Age -Stage	Univariate	DDFS* 5 years 78% vs 79% (*p* = 0.35)		5 years 79% vs 81% (*p* = 0.32)		MBC and TNBC stage I–IV: -70.5% and 77.7% AC -4.5% and 6.9% TC -2.3% and 0.8% FEC -0.0% and 0.8% docetaxel + carboplatin -13.6% and 11.5% paclitaxel + carboplatin -0.0% and 0.8% gemcitabine + cisplatin -0.0% and 1.5% gemcitabine + paclitaxel No significant differences in regimen use between MBC and TNBC. MBC and TNBC stage I–IV: -77.3% and 80.0% anthracycline received -13.6% and 13.1% platinum received -70.5% and 85.4% taxane received Significant difference in taxane use between groups (*p* = 0.041)
			Multivariate		DDFS* 1.64 (*p* = 0.22)		1.64 (*p* = 0.26)	

Abbreviations: *MBC* = metaplastic breast cancer, *TNBC* = triple negative breast cancer, *DFS* disease-free survival, *PFS* = progression-free survival; *OS* = overall survival, *HR* = hazard ratio, *CTx* = chemotherapy; *TAC* = taxane/paclitaxel, anthracyclines, and cyclophosphamide, *CMF* = cyclophosphamide, methotrexate, and fluorouracil, *FAC→TC* = fluorouracil, doxorubicin/anthracyclines and cyclophosphamide to taxane, paclitaxel/cisplatin/carboplatin, *AC/T* = doxorubicin and cyclophosphamide with/without paclitaxel or docetaxel; MAID = mesnex, doxorubicin, ifosfamide, and dacarbazine, *AC* = doxorubicin and cyclophosphamide, *TC* = cyclophosphamide and docetaxel, *FEC* = fluorouracil, epirubicin, and cyclophosphamide

Few studies have investigated immune-related marker expression in metaplastic and triple-negative breast cancers. One study demonstrated tumor cell PD-L1 expression in 32% of TNBC primaries and 40% of MBC primaries, though the expression was rarely strong and there were only five MBC samples. In addition, PD-L1 expression within the tumor-associated inflammatory cells was seen in 61.4% of TNBC primaries, and expression was maintained between 94% of matched primary-metastatic pairs [39]. Another study further demonstrated that PD-L1 expression was greater in MBC samples compared to TNBC (46% to 9%, *p* < 0.001) [19]. While our study showed similar results, Joneja et al. used a greater number of MBC samples to establish a better match between groups. There are no published studies that compared CD8 or CD163 expression in MBC to TNBC.

CD163 is a high-affinity scavenger receptor on monocytes and macrophages that binds to hemoglobin-haptoglobin complex and innate immunity sensor of bacteria [40]. It is regulated by pro-inflammatory and anti-inflammatory mediators and plays a role in many inflammatory diseases [41]. Elevated CD163 expression has been associated with lower survival rates among various cancers [42], including breast cancer [28, 43]. Another study showed that CD163^+^ macrophages in tumor stroma were positively correlated with certain pathologic characteristics seen in MBCs, such as higher grade, larger tumor size, and triple-negative/basal-like breast cancer [27]. While our study did not test for an association between CD163 expression and patient survival, our expression results show a numerical trend towards CD163 higher expression in MBC and are hypothesis-generating. These results can be further tested in future studies with larger patient numbers.

Unlike the expression of PD-L1 and CD163, CD8^+^ T-cells among tumor and stromal cells in TNBC are associated with a better prognosis and reduced risk of death [44]. Another study examined TNBC patients with high levels of CD8^+^ TILs and determined that greater levels of CD8^+^ TILs reduce the risk of recurrence and death [25]. Our study demonstrated reduced CD8 expression on immune cells in MBC samples (*p* = 0.02) in comparison to TNBC samples. These results suggest that future studies of immunotherapies for MBC could target tumor cell PD-L1, stromal CD163^+^ TAMs, or aim to increase the percentage of CD8^+^ immune cells. Future research could identify whether these markers act in a concerted manner to orchestrate immune suppression and if there is involvement from additional components of the tumor microenvironment.

The primary limitations of our study include its retrospective nature and the inclusion of a relatively small number of patients (*n* = 44), although this is consistent with sample sizes reported in previous studies, given the rarity of MBCs (see Table 5). As a result, more cases with longer follow-up could aid in improving the internal validity of this study. Additionally, our patient population is from only one large institution and predominantly white and non-Hispanic, which is not entirely reflective of the general population. Finally, the histologic types of MBC and TNBC samples were not identified for reasons including a lack of availability of tissue on all clinical samples.

## Conclusion

In summary, our data suggests that patients with MBC had similar outcomes to those with TNBC based on DDFS and OS. The use of taxane and anthracycline therapies was more common among our patients with MBC in comparison to another study examining this use among patients with MBC [34], although this was not independently associated with survival outcomes. Lower CD8, higher CD163, and higher PD-L1 staining in our MBC samples is comparable to data from previous publications using TNBC samples [25, 28]. Future studies are needed to confirm the prognostic role of tumor PD-L1, stromal CD163, and tumor CD8 in MBC, and further research is needed to see if these are potential therapeutic targets. MBC is a rare disease with a small patient population, so accrual to a prospective study remains a challenge for future studies to overcome.

## Data Availability

The datasets used and/or analyzed during the current study are available from the corresponding author on reasonable request.

## Abbreviations

CD163: Cluster of differentiation 163 protein
CD8: Cluster of differentiation 8 protein
CD8^+^: Cytotoxic T cell
DDFS: Distant disease-free survival
DFS: Disease-free survival
ER: Estrogen receptor
HER2: Human epidermal growth factor receptor 2
HR: Hazard ratio.
IC-NST: Invasive carcinoma of no special type;
M2: Alternatively activated macrophage
MBC: Metaplastic breast cancer
MRQ26: Ventana CD8 mouse monoclonal antibody
OS: Overall survival
OSU: Ohio State University
OSUCCC-James: The Ohio State University Comprehensive Cancer Center–Arthur G. James Cancer Hospital and Richard J. Solove Research Institute
PD-1: Programmed cell death protein 1
PD-L1: Programmed death-ligand 1
PR: Progesterone receptor
SP263: Ventana PD-L1 rabbit monoclonal primary antibody
SP57: Ventana CD8 rabbit monoclonal primary antibody
TAM: Tumor-associated macrophage
TME: Tumor microenvironment
TNBC: Triple-negative breast cancer
WHO: World Health Organization
ZL: Zaibo Li, MD, PhD
